# Advanced Applications of Polymer Hydrogels in Electronics and Signal Processing

**DOI:** 10.3390/gels10110715

**Published:** 2024-11-06

**Authors:** Ibragim Suleimenov, Oleg Gabrielyan, Eldar Kopishev, Aruzhan Kadyrzhan, Akhat Bakirov, Yelizaveta Vitulyova

**Affiliations:** 1 National Engineering Academy of the Republic of Kazakhstan, Almaty 050010, Kazakhstan; esenych@yandex.kz; 2 Department of Philosophy, V.I. Vernadsky Crimean Federal University, Simferopol 295007, Russia; gabroleg@mail.ru; 3 Department of Chemistry, Faculty of Natural Sciences, L.N. Gumilyov Eurasian National University, Astana 010000, Kazakhstan; kopishev_eye@enu.kz; 4 Department of Space Engineering, Institute of Communications and Space Engineering, Almaty University of Power Engineering and Telecommunication Named Gumarbek Daukeev, Almaty 050040, Kazakhstan; aru.kadyrzhan@gmail.com; 5 Department of Telecommunication Engineering, Almaty University of Power Engineering and Telecommunication Named Gumarbek Daukeev, Almaty 050040, Kazakhstan; a.bakirov@aues.kz; 6 Department of Chemistry and Technology of Organic Substances, Natural Compounds and Polymers, Faculty of Chemistry and Chemical Technology, al-Farabi Kazakh National University, Almaty 050040, Kazakhstan; 7 Department of Philosophy, al-Farabi Kazakh National University, Almaty 050040, Kazakhstan

**Keywords:** neuromorphic systems, digital signal processing, neural networks, multivalued logic, digital holograms, UAV groups, phase transitions

## Abstract

The current state of affairs in the field of using polymer hydrogels for the creation of innovative systems for signal and image processing, of which computing is a special case, is analyzed. Both of these specific examples of systems capable of forming an alternative to the existing semiconductor-based computing technology, but assuming preservation of the used algorithmic basis, and non-trivial signal converters, the nature of which requires transition to fundamentally different algorithms of data processing, are considered. It is shown that the variability of currently developed information processing systems based on the use of polymers, including polymer hydrogels, leads to the need to search for complementary algorithms. Moreover, the well-known thesis that modern polymer science allows for the realization of functional materials with predetermined properties, at the present stage, receives a new sounding: it is acceptable to raise the question of creating systems built on a quasi-biological basis and realizing predetermined algorithms of information or image processing. Specific examples that meet this thesis are considered, in particular, promising information protection systems for UAV groups, as well as systems based on the coupling of neural networks with holograms that solve various applied problems. These and other case studies demonstrate the importance of interdisciplinary cooperation for solving problems arising from the need for further modernization of signal processing systems.

## 1. Introduction

An approximation known as Moore’s law is often mentioned in the literature [1,2]. This approximation can be presented in various forms, but all of them, in essence, reflect the same fact: during the last decades, there has been a continuous increase in the number of logic elements per unit volume of semiconductor crystal (or rather, that was the goal of the chip makers). This trend sooner or later could not fail to come to an end for reasons known to be fundamental. The size of a “semiconductor” logic element cannot be reduced to a quite certain limit determined by the laws of quantum mechanics [3,4]. In a similar way, for elements whose size is comparable to the size of the interatomic distance in the crystal lattice, the concept of “electric current” loses its meaning: we can speak about the movement of a certain number of elementary charges.

These considerations are well known. As a consequence, there is currently a de facto active search for an alternative to the basis on which modern computing is built [5,6,7]. Not the least role here is played by research in the field of physics and physical chemistry of polymers, aimed at the development of a new basis for computing technology (more broadly, this is a means of digital signal processing). A very important example here is the developments in the field of information processing systems on what can be called a quasi-biological basis [8,9,10].

Here arises quite a certain contradiction, which, in our opinion, should be resolved in the spirit of the modernized theory of scientific revolutions, reflected, in particular, in [11]. Namely, modern computing systems are a special case of information processing systems (or, more precisely, signal processing systems, since the information with which computing operates can be represented only in the form of “signals” of one or another nature). Moreover, such systems use only one of the numerous types of logic—binary—although, in the 1960s, some unsuccessful attempts were made to create computing equipment based on ternary logic [12,13], the corresponding review is presented, in particular, in [14].

The obvious alternative is a neural network formed by the human brain; the nature of its functioning is very far from semiconductor computing. Modern computers, by means of which neural networks (as well as AI) are realized, are, in fact, a direct proof of the existence of the above-mentioned contradiction. With the help of programs embedded in computers, analogues of systems are realized in which signal processing is carried out on completely different principles. This thesis, in particular, is the basis for justifying the relevance of the development of neuromorphic devices of various types, which are being actively developed at present [14,15,16,17].

There is, however, an important nuance. Neural networks performing parallel computations can indeed be opposed to modern computer technology performing sequential computations. However, it does not mean that some or other “hybrid” devices occupying an “intermediate position” cannot be realized. Systems that utilize both the properties of typical electronic components and neural networks are also being actively developed at present; they are discussed in detail below. However, the “hybrid” nature of such systems does not affect the algorithmic basis: neural networks responding to such systems are oriented on the known algorithms of their training, and elements such as organic transistors are built on the basis of analogy with semiconductor elements oriented on the use of binary logic.

In our opinion, a review article should not necessarily be limited to the formation of some general picture of the results of research accumulated in a particular field of knowledge. There are situations when the amount of information accumulated in the literature allows us to reach a higher understanding of the problems under consideration only through their systematization and methodologically consistent interpretation.

This is the situation, in our opinion, that has now developed in the field of using the achievements of physics and chemistry of polymer hydrogels for the development of systems designed for digital signal processing. There are all prerequisites for realizing signal processing systems, not only built on a non-trivial element base but also using non-trivial algorithms of information processing. Moreover, there are more prerequisites to make the search for such algorithms purposeful (in particular, in order to maximize the possibilities of specific polymer materials).

This review, among other things, shows that for various purposes, it is reasonable to use digital signal processing systems built both on a different algorithmic basis and on a different element base. There is no need to reduce all such systems to either computing machines based on von Neumann architecture or to neural networks (or to their “hybrid” combination). In particular, it is acceptable to raise the question of using serial–parallel computations of various types.

## 2. From “Binary” Electronics to Polymer-Based Neural Networks

One of the main properties of polymer hydrogels is the ability to significantly change the degree of swelling due to external stimuli: changes in temperature [18,19,20], mechanical effects [21,22,23], ionic strength of the surrounding solution [24,25,26], electric current [27,28,29], etc. Such changes are often reversible, which allows polymer hydrogels to be used as a material for various kinds of sensors [30,31,32,33], including those for medical applications.

Sensor devices compatible with wearable electronics and implantable devices are of considerable interest [34,35,36], which fully utilize the increased sensitivity of hydrogels to external influences, as well as the biocompatibility of hydrogels. Devices of this kind are close to biointerfaces, which are used to convert signals of biological nature into electrical ones [37,38]. These devices can be considered as an example of systems that provide interfacing of systems using different algorithmic bases (e.g., the brain being diagnosed and the computer used for diagnostics).

An important example of such systems is biologically sensitive field-effect transistors (BioFETs), which are one of the most widespread classes of electronic sensors for the detection of biomolecules [39]. It is important to note that the interfacing of traditional electronic systems with biological systems should obviously take into account the difference in conduction mechanisms (in one case, the conductivity is electronic, in the other, it is ionic).

Problems of this kind are solved, in particular, by electrolyte-gated transistors (EGTs), i.e., devices with mixed ion–electron conductivity [40]. As emphasized in the cited review, electrolyte shielding provides significant advantages for the realization of neuromorphic devices/architectures, including ultra-low-voltage operation and the ability to form parallel-connected networks with a minimum number of wire connections.

The material of the review [40] also reflects one of the main trends that are emerging in the field of creating computing equipment based on functional polymeric materials. Namely, neuromorphic systems are understood as systems that, to a greater or lesser extent, imitate the operation of a neural network based on biological neurons. According to the authors [40], EGTs in the future allow for the realization of low-power, high-speed, and reliable neuromorphic computing for large-scale artificial neural networks (ANNs), intended, among other things, for the creation of new healthcare technologies in the form of an adaptable or trainable biointerface.

It is important to emphasize that the authors of the cited review consider the emulation of biological synapses using electronic devices only as a first step towards neuromorphic engineering and ANNs of this type. A similar point of view is shared by the authors of the review [41], who emphasize quite certain disadvantages of electronic computing machines based on von Neumann architecture. In particular, the spatial separation of the memory block and the computational processor leads to the necessity to provide continuous data movement between them. This, in turn, leads to significant time and energy consumption. As an alternative, in situ computing technology based on memristor architectures is considered. Memristors are non-volatile memory devices with multi-stage tunable resistance. Segnetoelectric (ferroelectric) polymers can serve as a basis for their realization, in particular, allowing for the creation of artificial synapses [41]. Polymer matrices of memristors capable of performing the functions of both arithmetic–logic elements, and accelerators of multiple accumulations for neuromorphic computation can be synthesized in various ways, including those providing the transition to nanoscale systems [42]; however, the general tendency to use analogies with the “natural” neural network formed by the human brain remains.

The results reflected in the above-cited reviews demonstrate a quite definite tendency characteristic of the developments in the field of the creation of new computing systems (more broadly, signal processing systems) based on new functional materials. Namely, this tendency is oriented towards borrowing this or that on an algorithmic basis, which has already justified itself when realized on a different “element base”. The use of logical elements, ultimately based on ideas dating back to Shannon, has justified itself in the form of modern computer technology, and neural networks, implemented in the form of computer programs, have also been proven to be the best. The borrowing discussed above does not have to be direct, and more often than not, it is not. Rather, it is a matter of finding relevant problems that can be solved by combining an algorithmic framework appropriate to “binary” computing with an algorithmic framework appropriate to neural networks.

Let us give additional examples illustrating the formulated thesis. Paper [43] considers the problem of sensory multitask learning aimed at creating systems analogous to the human retina and designed for pattern recognition. The authors rightly point out that the use of traditional silicon chips for this purpose is time- and energy-consuming. They have proposed a wearable dynamic system based on a sensor using, among other things, memory organic diodes, and the effectiveness of such a system has been proven on specific examples.

Another example is provided by [44], which also emphasizes that advances in organic transistors offer new opportunities for neuromorphic systems, focusing on such features of organic transistors as low-voltage operation and mixed ion–electron conductivity. Similar conclusions have been drawn in [45,46] on the development of artificial synapses.

It is appropriate to emphasize that the authors of [44] share the point of view of [47], according to which technologies based on the use of liquid-phase systems can be considered as an alternative to traditional processes for the production of complementary metal-oxide semiconductors (CMOSs), the grounds for which are created by successes in the field of chemical synthesis of new organic materials with predetermined (or specially selected) characteristics [48,49].

Returning to the thesis formulated above, it is appropriate to emphasize that many authors are of the opinion [44,50,51] that organic transistors used as the basis for the construction of neural networks (or their analogues) provide decentralized training on the corresponding carrier (crystal). From our point of view, the key word here is “training”. Namely, the actual algorithm according to which a neural network processes signals is not always known. Simplifying, we can express ourselves as follows. Algorithms of neural network training are well known and worked out, but the result of training is not always subject to unambiguous interpretation. It is this circumstance that led to research in the field of Explainable Artificial Intelligence (XAI) [52,53]. The task of this area is to provide verification and understanding of the results and outputs created by machine training algorithms. While traditional neural networks and deep learning (training) models are often seen as “black boxes” due to their complexity and lack of transparency, XAI aims to provide insights into how these models make decisions. This is particularly important in critical areas such as healthcare, finance, and autonomous driving, where understanding the rationale behind AI decisions is crucial for trust and accountability [54]. Explainable Neural Networks are a subset of XAI that focus specifically on providing explanations for the decisions made by neural networks [55,56,57].

The necessity to train neural networks (it does not matter on what physical and chemical basis they are realized), especially if we take into account the factor of their “logical opacity” (which forces the development of XAI), clearly illustrates the above thesis. Computers going back to Shannon’s ideas and any neural networks are built on a fundamentally different algorithmic basis. Moreover, there is also a difference at the philosophical level: in one case, we are talking about extremely transparent algorithms based on classical binary logic, while in the other case, we have to make special efforts to interpret the result of “training”.

Of course, it is possible to combine such systems, which is demonstrated by the materials of the reviews cited above. But it can be seen that in all the cases discussed above, it was about combining systems built on different algorithmic bases, but not about some synthesis of algorithms underlying their operation. On the basis of organic transistors or other analogues of electronic circuits, it is possible to realize a neural network, but (at least for now) such a neural network will have to be trained with the help of some algorithms.

Looking ahead, it follows from the main thesis formulated in the introduction that it makes sense to consider the prospects for the development of computing technology based on polymer materials, which will occupy an intermediate position between “binary electronics” and neural networks, with respect to the algorithmic basis. The prerequisites for such a statement of the question are also reflected in the current literature; they are discussed below.

For the time being, let us point out the works in which the trends mentioned above have been realized directly on the basis of polymer hydrogels. In [58], iontronic bipolar memristors having a three-layer polyelectrolyte gel structure were considered. The authors note that significant hysteresis of ionic current memristorization was successfully realized and the memristorization time was found to be geometrically scalable from 200 to 4000 s, and they emphasize that these memristors are versatile and provide enhanced functionality for neuromorphic computing. In [59], an ion exchange gel placed between the active layer and the aqueous electrolyte was used to switch the mode of operation of an organic transistor from that corresponding to an electrolytically controlled organic field-effect transistor (EGOFET) to the OECT mode.

There are also known reports responding to the trends under consideration, which utilize one of the main properties of polymer meshes—their ability to undergo reversible mechanical deformations. Thus, in [60], the issue of compatibility of stretchable rubbery electronics and sensors with biological organisms is considered. It is noted that the existing materials used for stretchable rubbery electronics are far from the degree of elasticity that, for example, mammalian skin, including human skin, possesses. In the cited work, fully polymeric rubbery transistors, sensors, and sensory skin approaching biological polymers are considered. In particular, all-polymer rubber transistors as well as all-polymer rubber strain and temperature sensors with high detection ratios are proposed. In [61], a similar problem was solved using polymer nanocomposite mesh frameworks assembled from polymer fibres.

Thus, the trends aimed at overcoming the shortcomings inherent in the “von Neumann architecture”, or, more precisely, inherent in computational technology, built on the principles dating back to Shannon’s ideas, can be realized, including on the basis of polymer hydrogels. Let us emphasize once again that from our point of view, there is no need to necessarily look for universal technical or algorithmic solutions. This statement is illustrated, in particular, by the works of [60,61]. Generalizing, we can assert that not only systems implemented on different physical and chemical bases, but also systems based on fundamentally different algorithms, can be used for different purposes.

This returns to the question of signal processing in biological systems. Indeed, neural networks, built on analogies with the functioning of the human brain, are the result of a very long evolution. There are also much simpler biological objects; there are also viruses, which, strictly speaking, occupy an intermediate position between living and non-living matter. All these objects, in accordance with the conclusions drawn in [62,63], can be considered, among other things, as information processing systems. More precisely, biological objects have a dual nature—they also have a quite definite informational “component”.

Consequently, if we start from general methodological considerations, then (from the point of view of modernization of computer technology) it makes sense to consider as “natural” analogues such information processing systems that do not necessarily belong to the higher forms of nervous activity too. The prerequisites for just such a statement of the question are also reflected in the current literature. In particular, there are numerous works in which attempts to realize logic elements on the basis of DNA and RNA are considered [64,65,66].

Further, ref. [67] considers the possibilities for creating chemical information systems in which the signal is formed through variations in the concentrations of chemical substances. This approach itself obviously corresponds to the information processing systems that are realized, for example, by viruses. Signals other than “chemically” in systems of this type (and even more complex ones) cannot exist. The electrical nature of nerve impulses is a product, let us emphasize it again, of a very long evolution. From the general methodological point of view, the concept of microchemomechanical systems considered in [67] is fully consistent with the above considerations. The functional elements in the considered systems are valves made of polymers capable of undergoing a phase transition. There is, however, a nuance. The authors of [67] considered their proposed microchemomechanical systems from the point of view of the possibility of realizing logical operations of the classical type.

Here, it is appropriate to emphasize that modern computer technology de facto tends to reduce all the operations that arise when trying to implement digital signal processing to the operation of addition and multiplication in terms of binary logic. We should recall that all modern computer technology is essentially built on Shannon’s fundamental idea: the keys connected in series perform the logical operation “AND”, and the keys connected in parallel perform the operation “OR” [68]. Any modern microcircuit, in fact, is an ordered set of such keys, supplemented with memory cells. The memory cells (triggers) are also assembled on binary logic elements.

A natural generalization of this approach is an attempt to use other types (including multivalued) of logic. In particular, computational systems based on DNA and RNA [69,70,71] need not necessarily lead to binary logic. It should also be noted that computational systems using multivalued logic [72,73,74], including those based on organic transistors [75], are currently under active development. Moreover, for interfacing classical logic elements with neural networks, the issue considered in [76,77] is of interest. Specifically, in these works, it was shown by methods of projective geometry that the operations performed by neurons with a threshold activation function can be reduced to logical ones. In the future, the development of such an approach will make it possible to remove many problems associated with the logical opacity of ANNs. Consequently, there is good reason to include in this review a brief consideration of algorithms for computing systems oriented to the use of multivalued logic.

## 3. Variability in the Physicochemical Foundations of Computational Science and Their Complementary Algorithms

The modern “digital” world is built on binary logic, based on two fundamental facts, one from the field of mathematical logic, and the other from the field of technical physics. In mathematical logic, it is proven that any binary logical operation can be reduced to two others [78]. Shannon’s idea, mentioned above, makes it possible to realize the operations “AND” and “OR” by electrical means, with the help of keys closing and opening the electric current, and, therefore, any other operations of binary logic.

These facts are generally known, but based on the methodology used, they can be viewed from a point of view somewhat different from the generally accepted one. The success of the “binary digital world” was guaranteed by the fact that its logical–mathematical and physical–chemical (physics and chemistry of semiconductors) foundations turned out to be complementary to each other. Consequently, if it is proposed to use a computing technique built on a non-traditional physical and chemical basis to solve this or that ranges of specific problems, it is reasonable to raise the question of finding an adequate algorithmic basis at once.

On this basis, it is necessary to make a digression into the realm of number theory and multivalued logic. The basis of all modern computing is operations on integers represented in binary form. Formally, the range of their variation is infinite; however, real computer technology always deals with integers changing in a finite range. Thus, a 16-bit processor operates with numbers not exceeding 2^16^.

This fact, among others, means that the algorithmic basis of any computational technique will be oriented to finite sets. Consequently, any algebraic structures containing a finite number of elements (Galois fields [79], finite algebraic rings [80], etc., which find a variety of practical applications, in particular, in cryptography [81,82]) can be used to represent integers. As noted in [83], in the natural science tradition, physical phenomena are, as a rule, described by functions that take real or complex values. However, as emphasized in [84,85], this is nothing more than a matter of agreement. A function that takes values in a particular set is nothing more than a model of a real process such as, for example, a signal [84,85]. From a general methodological point of view, there is a mapping of a real physical process (for example, signal generation) onto a certain mathematical object, the choice of which is ultimately determined by issues of convenience and efficiency of use. One of the well-known examples of finite algebraic structures is the residue number system (RNS), which can be used to significantly increase the efficiency of computing devices [86,87], and this also applies to neural networks [88,89]. In the construction of an RNS, modulo integer computations are used (the term modular arithmetic is also used [90,91]).

Further, there are important examples of multivalued logic that can be reduced to computations in Galois fields, traditionally denoted as GF(p) where p is a prime number. For this purpose, it suffices that the number of values that the variables of a multivalued logic take is equal to p [92]. It should be noted that the traditionally used binary logic can be viewed as computations in the field of GF(2). It is also possible to reduce the operations of p−1-logic to computations in algebraic rings [93].

Let us emphasize that the transition to modular arithmetic automatically implies performing calculations on modulo integers. Such operations can be realized by means of logic elements corresponding to binary logic, but this approach is far from being optimal. Operations with each “component” of an integer represented in the RNS actually represent computations in a Galois field of the simplest type. The operations in such fields, in turn, can be put in correspondence to the operations of multivalued logic. Consequently, the transition to an element base that fully corresponds to the use of modular arithmetic cannot be associated with the performance of operations in terms of multivalued logic. Consequently, the next step in the development of computing systems overcoming the shortcomings of existing computers will inevitably be oriented to the use of an element base corresponding to various variants of multivalued logic (which, of course, does not imply a complete rejection of the use of binary logic). It is from this point of view that polymer-based systems are of particular interest.

We emphasize that systems, adders, and multipliers of modulo integers, which can be used for computations in Galois fields, and hence for performing operations of multivalued logic, have also been actively developed for quite a long time [94,95,96]. When operations are performed in terms of p-logic, the question of memory cells complementary to such logic arises. In typical electronic circuits, memory cells are assembled on the basis of RS triggers and their analogues. These are systems capable of being in two different stable states, one of which can be set to correspond to a logical zero and the other to a logical one. Any physical–chemical system capable of being in two different stable states can serve as a binary memory cell too. In particular, memristors mentioned above and systems based on hydrophilic polymers experiencing phase transitions accompanied by hysteresis phenomena [97] can be used for this purpose. Similarly, systems based on hydrophilic polymers experiencing phase transitions accompanied by hysteresis phenomena can be used for memory cells corresponding to the p-logic, and systems based on hydrophilic polymers, in which the phase transition is carried out in several stages, can be used; the corresponding review is presented, in particular, in [98].

Specifically, in the cited work, using the phase portrait method, it was shown that the curves reflecting the nature of the phase transition can be dissected into several sections, each of which corresponds to a fragment of either a parabolic or linear phase portrait. On this basis, it can be concluded that there are examples of polymer-based systems in which phase transitions occur in several stages, which is expressed by the term “stadial phase transition” used hereafter.

The existence of stadial phase transitions, in its turn, in the future allows us to move to the formation of neural networks complementary to the p-logic. Moreover, analogues of such networks can spontaneously form in solutions of hydrophilic polymers [99,100]. As shown in the cited papers, macromolecular coils present in solution are affected by the surrounding coils. The nature of this influence can be different. Thus, in sufficiently concentrated solutions, individual fragments of one macromolecule can penetrate into a coil formed by another. If a polyelectrolyte solution is considered, the coils can influence each other due to the effect of redistribution of concentrations [101], etc. There is also the effect of remote interaction found in macroscopic samples of hydrogels [102,103]. The authors are of the opinion that the same effects can also occur in relation to the interaction of macromolecular coils, the sizes of which significantly exceed the Debye length. Indeed, the mechanism of remote interaction of hydrogels is as follows [104,105].

Let us consider two cross-linked polymer networks placed in a solution of a low-molecular-weight salt. One of these networks acts as a proton donor (e.g., it can be a network based on cross-linked partially neutralized polyacrylic acid) and the other as a proton acceptor (e.g., it can be a network containing amine or imine functional groups). As a result of proton binding by the proton acceptor gel, it would have to acquire an additional electrostatic charge, and the gel donor would have to acquire a charge of the opposite sign. However, these charges act on the low-molecular-weight salt ions in the solution above the gel. As a result, ions of one sign are bound by the gel donor and others by the gel acceptor. This effect can obviously be applied, among other things, to the extraction of ions from solution [104,105]. It can also be seen that in order for the separation of low-molecular-weight ions to take place, it is sufficient that their localization regions do not overlap due to the thermal motion of the ions. Consequently, the effect under consideration will also take place in the case when, instead of gels, any other objects whose dimensions exceed the Debye length, such as macromolecular coils, are considered.

**Figure 1:**
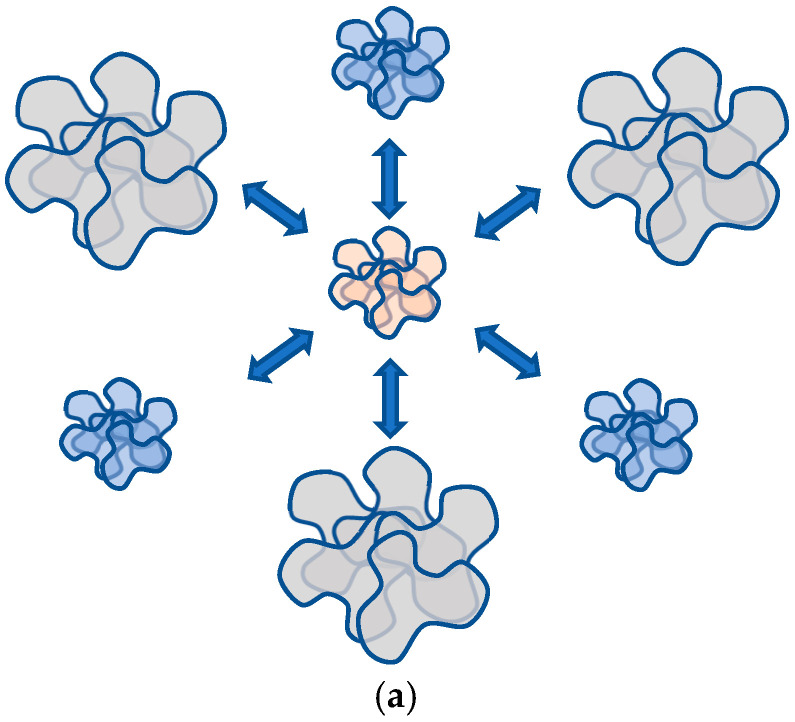

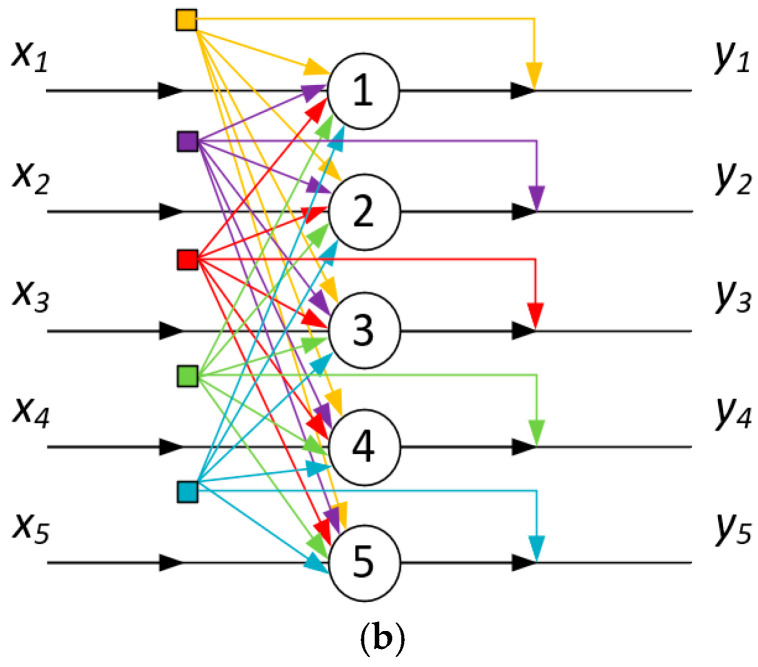
Generalized scheme of neural network analogue formation in hydrophilic polymer solutions (**a**); Hopfield neural processor scheme (**b**) [99].

Let us take into account that this remote interaction effect is reversible; hence, an analogue of a neural network based on the scheme of Figure 1a can indeed be realized (provided that the gel contains not only ionogenic functional groups but also groups leading to the appearance of a phase transition [99,100]). Such a network is a direct analogue of a Hopfield neural processor (Figure 1b).

Let us make a clarification: hydrophilic systems can be understood as any system whose behaviour is somehow determined by the nature of interaction with water. In such systems, of course, hydrophobic interactions can be expressed; moreover, their properties are often determined by the hydrophobic–hydrophilic balance. Strictly speaking, it is acceptable to use the term “hydrophilic–hydrophobic systems”, but hereafter, we will stick to the term “hydrophilic”.

We emphasize that hydrogels of various compositions have been obtained to date, including those that simultaneously contain both ionogenic and hydrophobic functional groups, i.e., ionogenic gels can be made thermosensitive [106,107]. Consequently, the possibility of creating an analogue of a neural network based on gels that enter into remote interactions by the mechanism discussed above is quite real.

However, the following fact is of much greater interest. Suppose that a polymer coil, which, according to the scheme of Figure 1, plays the role of a separate neuron, experiences a phase transition accompanied by hysteresis phenomena. Such a situation, as follows from the materials presented above, is quite real. In this case, at least, we can assert that the analogue of a neural network of this type has two forms of memory—both distributed (i.e., characteristic of a neural network of the classical type) and concentrated (i.e., that which corresponds to the creation of individual memory cells in modern computers). An individual ball, the phase transition which is accompanied by hysteresis phenomena, is itself capable of forming a “memory cell”, as noted above.

The situation becomes even more complicated when phase transitions accompanied by hysteresis phenomena become stadial [98]. In this case, the analogue of a neuron turns out to be capable of being in several different stable states, which corresponds to the p-logic. Moreover, if the stadial phase transition is also accompanied by hysteresis phenomena, then the memory cell corresponding to a single macromolecular coil also corresponds to this logic.

A neural network in which local memory cells are embedded is more than a specific object. At a minimum, it can be argued that the response of such a system to external influences will depend not only on the current state of the network but also on the exact path along which this state was created. This fact seems more than important from the point of view of “training” neural networks created on a non-trivial physical and chemical basis. Indeed, the “training” of a neural network in the traditional sense of the term is related to changing the weight coefficients of the network [108]. In the case of physicochemical systems, in particular, those based on the use of polymeric macromolecules, the possibility to control the values of weight coefficients is not always available. It can be argued that there are situations when the effective values of weight coefficients are determined by the structure of the material itself.

The simplest illustration of this thesis is the analogues of neural networks that can be constructed on the basis of inhomogeneous hydrogels. Namely, proofs of the existence of analogues of neural networks formed by hydrophilic polymers were given in [98,99] on the basis of the consideration of solutions. At present, methods of obtaining inhomogeneous polymer networks have been developed [108,109,110]. Based on the analogy with the behaviour of inhomogeneous solutions, it can be argued that it is possible to obtain an analogue of a neural network on the basis of polymer hydrogels, in which the role of individual neurons will be played by sections of the gel that differ from neighbouring ones in their physical and chemical characteristics (for example, in the content of hydrophobic groups that determine the property of sensitivity to temperature variations [111,112]).

Obviously, in such a system, the degree of influence of one of the sections of the polymer network on the others will be completely determined by its structure; accordingly, the possibilities for reconfiguring the weight coefficients of the considered neural network analogue are, to put it mildly, limited.

The analogue of a neural network, in which local memory cells are embedded, allows us to overcome difficulties of this kind. Indeed, as noted above, the state of hydrogels, generally speaking, is determined by a set of thermodynamic variables, and situations can be realized when cross-linked polymer networks turn out to be susceptible, among others, to magnetic fields [113,114] and to optical range signals [115,116]. This fact proves that the range of stimuli effects on hydrogels can be quite wide.

**Figure 2:**
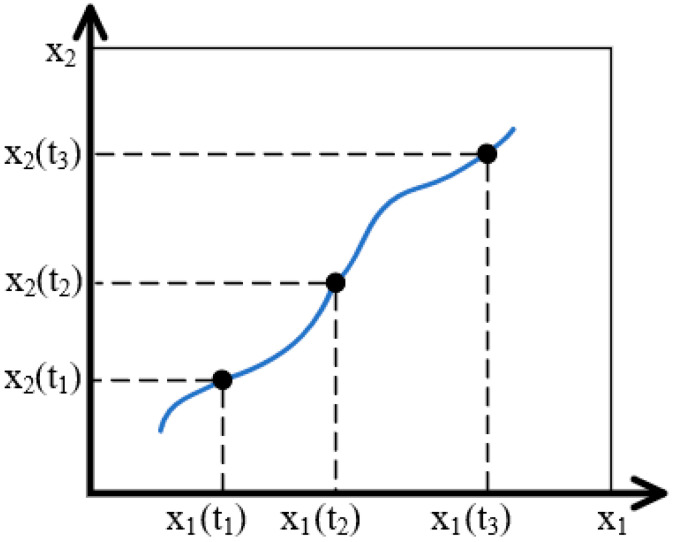
An illustration of the concept of “image point”.

For clarity, we will use the case when the hydrogel-based neural network analogue is described by variations in two thermodynamic variables $X_{1,2}$. In this case, the variation in external conditions can be reflected by an image point that moves along a two-dimensional map, as schematically represented in Figure 2.

**Figure 3:**
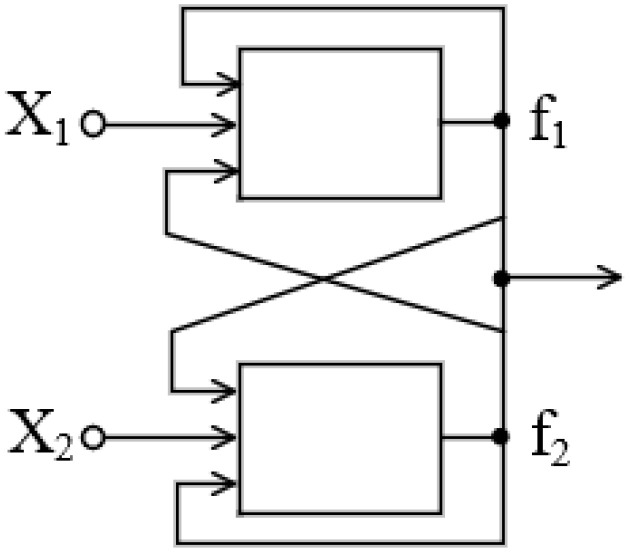
A scheme of the analogue of a trigger on two neurons with a sigmoidal activation function.

Based on the analogy with the existence of hysteresis phenomena, it is acceptable to assume that a neural network containing “embedded” memory cells can be in several different stable states at the same values of control parameters. This conclusion has been proven, in particular, by the simplest model presented in [117]. In a cited report, an analogue of the RS trigger, the circuit of which is shown in Figure 3, is considered.

It is assumed that each of the elements of this scheme is similar in properties to formal neurons used for the synthesis of artificial neural networks (ANNs) and is described by a sigmoidal activation function

(1) $$f\left(x\right)=\frac{e^{\alpha x}-e^{-\alpha x}}{e^{\alpha x}+e^{-\alpha x}}$$

where

(2) $$x=w_{1}q_{1}+w_{2}q_{2}+w_{3}q_{3}$$

Here, $w_{i}$—weighting coefficients; $q_{i}$—variables describing the state of neuron inputs; and α—constant coefficient for the given scheme.

In the simplest case, when the connection is symmetric and the weight coefficients corresponding to the feedback in a given circuit are the same, the analogue of the trigger under consideration is described by the following system of equations:

(3) $$\left\{\begin{array}{c} f_{1}=f\left(z+x_{1}\right)\\ f_{2}=f\left(z+x_{2}\right)\\ z=k\cdot \left(f_{1}+f_{2}\right) \end{array}\right.$$

Variables $x_{1}$ and $x_{2}$ in Formula (3) can take values ranging from −1 to +1.

**Figure 4:**
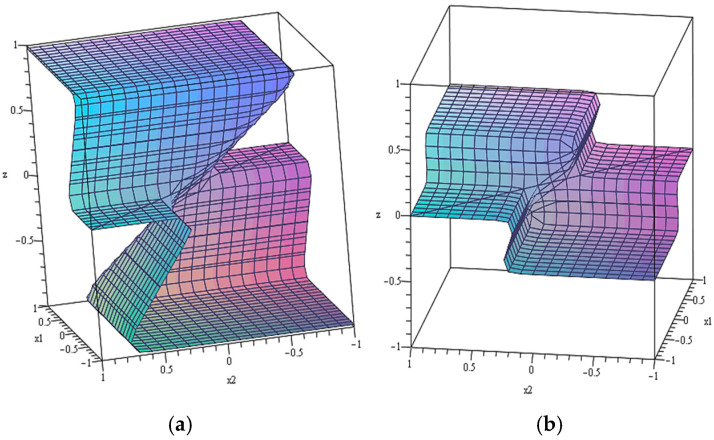
A three-dimensional graph of the dependence of the solution z of the system of Equation (3) at the different values of variables $x_{1}$ and $x_{2}$: α=10, k=0.49 (**a**); α=10, k=0.2 (**b**) [117].

An example of the numerical solution of system (3) is presented in Figure 4.

**Figure 5:**
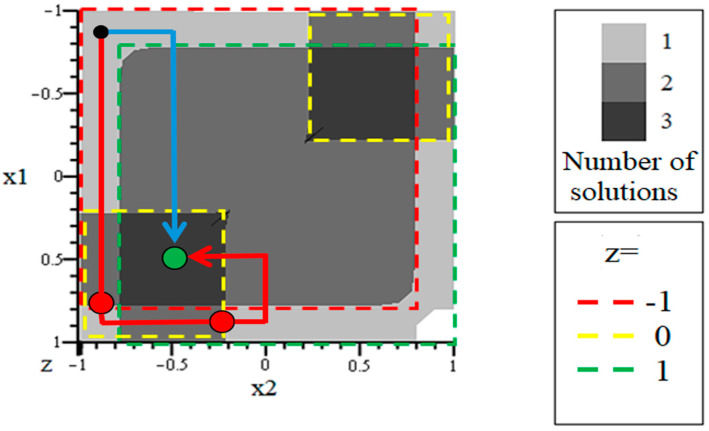
Decision map for the considered tax trigger based on two neurons; parameters: *a* = 10 and *k* = 0.5 [117].

It can be seen that the dependence of the system state on the control parameters corresponds to a multivalued function. This, in particular, means that at the same values of thermodynamic variables $X_{1,2}$, the system under consideration can be in several different states. Moreover, the realization of a particular state of the system depends not only on the current values of $X_{1,2}$, but also on the trajectory of the representing point on the map, schematically represented in Figure 2. This follows from the fact that the folded surface obtained even for the simplest model has a rather complex shape, which leads to a kind of “multidimensional hysteresis”. Accordingly, different trajectories of the imaging point can lead to different states of the system, as demonstrated by Figure 5 [117].

The darkest colour in this figure shows the areas that correspond to three possible solutions of the system of Equation (3). The lightest colour shows the areas that correspond to only one possible solution. The intermediate colour corresponds to two solutions. In this figure, the dashed lines also show the areas that correspond to the three possible solutions of the system of Equation (3). z≈−1, z≈0, and z≈1.

Figure 5 shows two possible trajectories of the imaging point. It can be seen that at the same initial and final values of a pair of control parameters, the point can move along different paths. The trajectory shown in red corresponds to two jump transitions shown by the bold red dots. One of these transitions is from the state z≈−1 to the state z≈0 and the other is from the state z≈0 to the state z≈1 which corresponds to the end point of the trajectory. When moving along the trajectory shown in blue, there are no transitions, i.e., when reaching the end point of the trajectory, the system remains in the state z≈−1.

The obtained result, among other things, illustrates how exactly the analogue of a neural network can be “trained”, possessing a rigid structure, which does not allow us to raise the question about variations in weight coefficients at all. Namely, each specific trajectory of a representing point in a thought experiment can be divided into two parts. The first part can be correlated with the prehistory of the system (i.e., with those influences that were applied to it before the “zero” point in time), and the second part with the predicted response to additional external influences.

**Figure 6:**
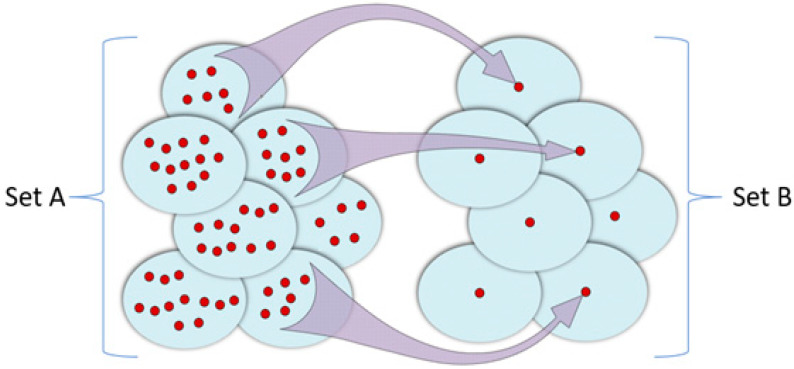
A morphism of set A into set B defining a partition of set A into subset $A_{i}$, each of which corresponds to a particular code combination with no error [118].

It remains to be considered to what extent neural network analogues with rigid link architectures (which exclude changes in weighting coefficients) can really be likened to neural networks of the classical type. This question is answered by the results of [118], in which it was shown that analogues of neural networks can be realized based on the analogy with the technique of constructing error-corrected (noise-resistant) codes. Indeed, the functioning of a neural network that does not contain feedback (e.g., a feedforward neural network) can be described through a morphism, which is illustrated in Figure 6. This morphism also corresponds to the basic idea of the use of noise-resistant codes, the best known of which are the Hamming codes [118]. Indeed, in accordance with the noise-resistant coding technique, in the original digital sequence,

(4) A=(0,1,1,0,0,…,0,1,1,0)

which are additionally entered characters associated by a certain rule with the characters contained in the original sequence.

(5) $$A\to A^{+}=(0,1,1,0,0,\ldots ,0,1,1,0,a_{1}^{+},\ldots ,a_{n}^{+})$$

This redundant information allows us to reconstruct the initial code in the same way as redundant information contained in messages in natural languages allows us to reconstruct their meaning from the context. The work of Hamming codes can be reflected in the diagram shown in Figure 6.

Set A of all possible code combinations is divided into subset $A_{i}$, the number of which is equal to the number of code combinations treated as code with a corrected error. Any $a\in A_{i}$ is matched with a code combination with a missing (or corrected) error from set B. The procedure of image recognition by a neural network can be considered from exactly the same positions. The inputs of neurons forming the first layer of the network are fed with a set of binary variables, interpreted as a recognized image, possibly containing errors. At the outputs of neurons of the last layer of the network, a set of signals is formed, which constitute in the aggregate the original image, which does not contain errors.

For this situation, the scheme in Figure 6 is also applicable [118]. Elements of the subset to which the mapping is performed can be treated as recognizable images, and elements of the original set can be treated as images containing errors. This analogy makes it possible to identify quantitative patterns that characterize neural networks of various structures.

It should be noted that the results of [118] clearly correlate with the results of [76,77], in which, as noted above, it was shown that the response of a neuron possessing a threshold activation function can be reduced to logical functions. Moreover, the nature of surfaces similar to Figure 5 becomes significantly more complicated in those cases when the controlling thermodynamic variables are more than two. More precisely, in this case, we should pass from surfaces in three-dimensional space to hypersurfaces in multidimensional space. The authors are of the opinion that in such a case, several stable solutions may correspond to the same set of control parameters.

This is another basis to further move towards the use of multivalued logic for the development of polymer hydrogel-based computing systems. There is an important nuance in this formulation of the question. Namely, neural networks or their analogues, realized on the basis of polymer hydrogels, in many cases, will obviously lose in speed to systems based, for example, on nanostructures, which are also being actively developed at present [119,120,121]. At the least, the speed of ion movement in the hydrogel volume is orders of magnitude lower than the speed of electron movement, including that along nerve fibres.

There is, however, quite a definite class of problems, closely related to the problem of signal processing, for which the above-mentioned fact is not fundamental. Specifically, we are talking about the potential use of systems based on hydrogels to provide information protection when transmitting messages in the zone of direct radio visibility, as well as to prevent the detection of aircraft by radar. Consideration of these issues is of interest, among other things, because systems of the type discussed below can have relatively low speed (at a level corresponding to the audio frequency range). They also do not require high-precision manufacturing (the characteristic size of such systems is comparable to the wavelength of the gigahertz range). At the same time, they make the implementation of neural networks very interesting, of which the characteristic size of elements can be several millimetres and more, which is very convenient for experimental studies, as well as from the point of view of visibility.

## 4. Metamaterials Based on Polymer Hydrogels: Prerequisites for Practical Applications in Signal Processing

Currently, metamaterials and metasurfaces, including those obtained using polymer hydrogels, attract a steady interest from researchers [122,123,124]. Metamaterials are matrices (e.g., polymeric) containing additional inclusions (e.g., made of metal in the shape of the letter “omega”, or derived from graphene, etc.) [125,126,127]. Metamaterials can be considered as artificially created composite materials possessing a negative refractive index value, which leads to their very non-trivial interaction with electromagnetic oscillations, including those of the optical range. In particular, a plane-parallel plate made of a metamaterial can fulfil the functions of a lens [128,129,130].

Inclusions (metaatoms), which interact resonantly with electromagnetic vibrations (which provide a negative value of the refractive index), can be fabricated in various ways [131,132,133], but the geometry of their arrangement also plays an essential role. This is why hydrogels are of significant interest for the fabrication of metamaterials [134,135]. It is possible to control the characteristics of metamaterials using radio [136] or optical [137] signals. Metamaterials have a wide range of advanced applications; however, it makes sense to focus on a quite certain private problem, which is closely related to the ability of metamaterials to controllably transform electromagnetic radiation (in this case, due to geometric factors, the most convenient is the radio range). This problem is closely related to one of the obvious applications of metamaterials—providing cloaking in both the radio and optical ranges. It is appropriate to emphasize that technologies solving such a problem in the radio range (Stealth) have been developed for quite a long time [138,139]. To realize the Stealth technology, it was proposed to use materials that absorb radio waves [140,141], as well as programmable reflection, scattering, etc.

There is no doubt that the use of metamaterials creates quite certain prospects for the improvement of such technologies. A certain part of the aircraft surface can be transformed into a set of lenses, making it impossible to identify its coordinates accurately. Technologies based on such principles, in the future, can significantly transform the existing approaches to information protection, at least in the area of direct radio visibility.

The relevance of information protection during transmission over relatively short distances is determined by the following factors. At present, there is a steadily increasing interest in the use of groups of unmanned aerial vehicles (UAVs) [142,143,144] that, in the future, can be capable of operating in autonomous mode (including target search, etc.). Algorithms providing control of UAV groups have been considered in many earlier works [145,146,147], but the current situation prompts one to search for non-trivial solutions to the corresponding problems.

It follows from the most general considerations that a group of UAVs will represent a systemic whole if and only if all its physical components (vehicles) exchange signals with each other. In the future, such a group may well become a carrier of distributed artificial intelligence, built on a detached analogy with the human brain. For the realization of such a system, the problem of information protection is of decisive importance. As a rule, it is solved by using cryptographic methods [148,149]. The current literature, however, also reflects an alternative approach focused on the use of physical processes [150,151,152]. In the limiting case considered in [153,154], information protection can be ensured by identifying the point in space to which the source of a signal interpreted as “one’s own” is located.

This approach, of course, can only be used in the direct radio line of sight, but this is precisely the situation that corresponds to the use of groups of UAVs capable of operating autonomously in the future. Accordingly, each UAV should ideally be equipped with an antenna system that allows the spatial position of the signal source to be determined. (In the works cited above [153,154], the role of such an antenna system was played by the UAV group as a whole.)

There is no doubt that the use of metamaterials (metasurfaces) can significantly affect the nature of solving problems of this kind. Indeed, there is a (at least theoretical) possibility to transform almost the entire surface of a UAV into an analogue of an adaptive optical system tuned to a certain spatial position of the signal source. Moreover, the very nature of information exchange between UAVs forming a group with AI installed on it can be significantly transformed. When using metamaterials and metasurfaces that provide the formation of focusing systems in the centimetre wavelength range, the signal transmission from one UAV to another can be converted into a passive form.

**Figure 7:**
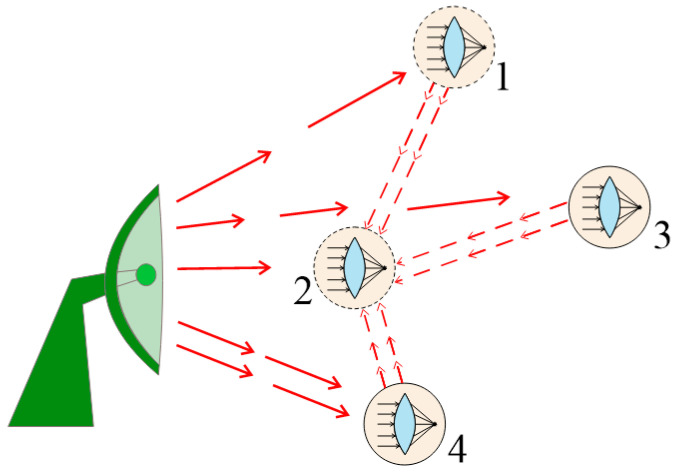
An illustration of the possibility of using metamaterials to control groups of UAVs.

The scheme of information transmission in such a mode is presented in Figure 7. At a certain time interval, the focusing system installed on the UAV with number 1 focuses the signal transmitted from the operator to the UAV with number 2. The other elements of the group perform a similar operation during this time interval. In this approach, the entire group of UAVs (except for the UAV with number 2) works as a focusing system, which allows us to not only identify the operator’s location but also to ensure the transmission of control signals.

Further, the same approach can be used to transfer information from one element of the group under consideration to another. Indeed, if we use binary encoding, it is enough to provide focus modulation. At some clock cycles corresponding to the sequence of transmitted ones and zeros, focusing takes place, at others, focusing does not. This example, of course, is mainly hypothetical in nature. Nevertheless, it clearly demonstrates the essence of the basic thesis of this review.

The necessity of using high-performance chips that complete UAVs is mainly due to the use of cryptographic methods to protect information. The number of commands that actually need to be transmitted to the vehicle is obviously small [155,156], so the device that controls the vehicle can be configured for a fairly low data rate (within the range of tens of Hertz). Such a frequency of change in the characteristics of metamaterials is quite achievable even at the current level of research in this field [136,137].

The considered example shows that the focus on a further increase in processor technology performance corresponds only to one of the directions of development of signal processing means. There are also other options, and the choice of a particular approach turns out to be organically connected with the specifics of the range of tasks to be solved.

It is pertinent to emphasize that the issue of using metamaterials precisely as transducers of electromagnetic oscillations used for information transmission is also attracting a growing interest of researchers, and this applies to the optical [157,158] and radio bands [159,160]. One of the non-trivial potential applications in this respect is related to radio holography, which is also quite actively developed at present [161,162,163]. As emphasized in the cited works, the fundamental difference between radio holography and optical holography is the fact that in radio holographic systems it is possible to fix the phase of electromagnetic oscillation. The same is true for hologram analogues developed for acoustic oscillations [164,165,166]. This greatly simplifies the fabrication of analogues of holograms used in the radio band. Such analogues, among others, can be fabricated on the basis of metamaterials.

Indeed, any hologram can be considered a radiation converter, and if we are talking about monochromatic radiation, it is acceptable to speak about wavefront converters [167,168]. The effect of a metamaterial with distributed characteristics on monochromatic electromagnetic radiation can be described through its equivalent optical scheme, which is reduced to a set of tunable lenses or parabolic mirrors. This conclusion is general and follows from the peculiarities of solving the problem of propagation of monochromatic electromagnetic oscillations with certain boundary conditions [169,170]. As shown in the cited works, any transformations of the local section of the wavefront are driven by transformations realized by a thin lens or a parabolic mirror. Metamaterials are no exception in this respect; the only difference is that it is required to take into account the peculiarities of boundary conditions at the boundaries of media with positive and negative refractive index values [125,128].

The fact that analogues of holograms (both for electromagnetic and acoustic oscillations) can be realized on the basis of metamaterials, which, in turn, represent a physical realization of neural networks (which follows from the materials presented above), opens up very tempting prospects for the development of systems designed to process not just signals, but to process waves possessing a rather complex spectrum of spatial frequencies. We emphasize that a “signal” usually corresponds to a function depending on a single variable (a time variable). If, however, an electromagnetic or acoustic monochromatic wave is used, it is described by a function depending on two variables. This corresponds to the fact that the wave can provide the realization of a certain set of “one-dimensional” data transmission channels, which is used, in particular, in fibre-optic communication lines.

Thus, it can be stated that metamaterials allow us to realize a certain “hybrid” of radio or acoustic holograms with neural networks. This creates quite certain prospects for recognizing three-dimensional images diagnosed by means of acoustic or radio holography methods. A typical task for acoustic holography is the task of diagnosing the state of human internal organs. Similar tasks arise in the radio range; in particular, they are associated with the diagnosis of subsurface objects (ore bodies, underground caves, etc.) [171,172,173].

The solution to problems providing information about the nature of subsurface objects (or the state of human internal organs) can be provided by computational means or means of visual display of information (as is the case in modern devices for ultrasound diagnostics). Further improvement of metamaterials, including those based on polymer hydrogels, allows us, however, to approach the solution of this problem from a slightly different side, fully corresponding to the possibility of interfacing metamaterials with neural networks (or their analogues).

**Figure 8:**
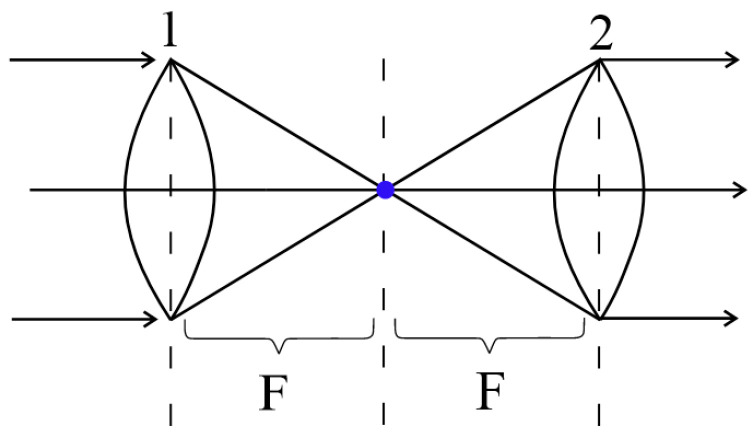
Classic telescopic system.

Let us consider for illustration a telescopic system formed by two identical lenses (Figure 8). In such a system, the focal points of the first and second lenses are aligned at the same point. This means that any plane wave arriving at such a system will also be transformed into a plane wave.

Returning to advanced applications of metamaterials, the optical scheme shown in Figure 8 can be looked at from a slightly different point of view. Namely, this scheme has a primary wavefront transducer (lens 1) as well as a complementary one (lens 2). On this basis, this scheme can be generalized.

**Figure 9:**
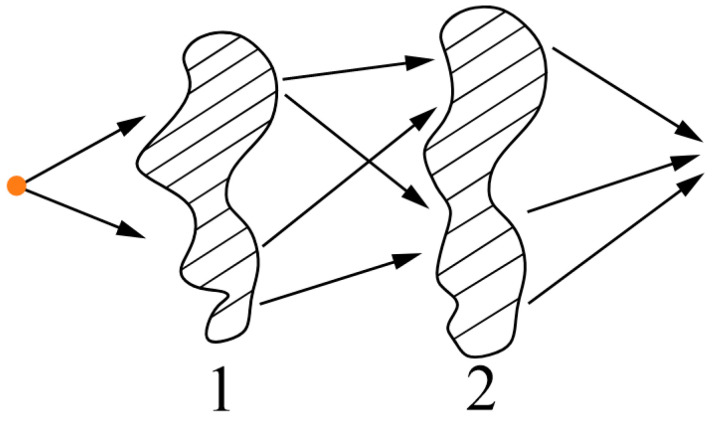
Forward and backward radiation transducers as a means of diagnosing objects inaccessible by direct contact.

There is an object to be diagnosed, which in one way or another transforms the incident monochromatic radiation (1, Figure 9). There is another object realized on the basis of a neural network based on a tunable metamaterial (2, Figure 9). It follows from the general theory of wavefront transformation of monochromatic radiation, which was reduced to its discrete form in [168], that for an arbitrary wavefront transducer, there must exist a wavefront inverse to it (which correlates with the classical principle of ray reversal).

Consequently, diagnostics of a three-dimensional object inaccessible for direct contact (patient’s internal organs, subsurface objects, etc.) when using metamaterials providing realization of neural networks can be based on the pairing of a “secondary” transducer with a “primary” one. Verification of such conjugation, among other things, can be carried out according to the scheme of Figure 9. If the “secondary” object provides focusing in a certain point of space, it can really be considered complementary to the “primary” one.

This approach allows for building objects that are complementary to the recognized one, and all the above-mentioned developments related to the formation of neural networks based on hydrophilic polymers can be used here. An obvious advantage in this respect is the fact that the fabrication of a “secondary” radiation transducer does not face strict requirements on dimensions, geometry, temperature regime, etc. Moreover, the circuit in Figure 9 fully meets the basic ideas behind the creation of neuromorphic devices. A “secondary” radiation converter fully utilizes the basic advantage of neural networks—it performs parallel processing of the whole set of “one-dimensional” signals, which is carried by radiation possessing a certain wavefront.

There is quite a wide range of possibilities for the realization of a “secondary” radiation transducer. For example, it can be realized on the basis of computational techniques that are already being used in radio holography to establish the shape of the object under study. It is also possible to use the techniques actually used by convolutional neural networks when the image of the object is constructed from elements of geometric figures or lines. A combination of these and other methods can also be used.

The advantage of schemes similar to the one presented in Figure 9 is that they allow us to directly verify the compliance of the calculation results (or empirical selection) with the real characteristics of the object under study. It is also significant that the above-mentioned properties of analogues of neural networks, including those based on hydrophilic polymers, make it possible to realize the scheme of Figure 9 or something similar using both electromagnetic waves of various ranges and acoustic vibrations. The application of the latter for medical diagnostics is obvious.

Applications of holograms conjugated with neural networks (or their analogues) also open up many other perspectives. In particular, if in accordance with the scheme in Figure 9, it is possible to realize a radiation transducer complementary to the initial object, then further on the same algorithmic basis, it is possible to realize another holographic transducer, which will already restore the volumetric image of the object under study. For example, the next step in the development of the means of ultrasonic medical diagnostics is to realize a volumetric (holographic) image of diagnosed internal organs.

There is no doubt that the conjugation of neural networks with holograms, realized, including those based on polymer hydrogels in the foreseeable future will create all necessary prerequisites for the realization of such kinds of systems. It should be emphasized that the advantages of hydrogels in this respect are associated, among other things, with the implementation of “thick-layer” holograms that allow us to reproduce volumetric colour images and process them in parallel.

However, a detailed analysis of such possibilities is beyond the scope of this review.

The main conclusion that can be made on the basis of the presented (albeit far incomplete) analysis of the situation that has developed in the field of development of innovative systems based on the use of organic materials and designed for signal and image processing can be formulated in the following form.

Exhaustion of the potential for further development of computing technology, built on the classical semiconductor basis, obviously implies the search for alternative possibilities. Possible variants are reflected in the current literature analyzed above, and they are characterized by a very significant diversity. Based on the general provisions of the theory of scientific revolutions [11], it can be argued that the development of computing technology (more broadly, the technology designed for signal and image processing) is currently at a certain “bifurcation point”, since there is a very large variety of physical and chemical systems on which can be realized promising signal processing systems, as well as their algorithmic foundations. At a certain stage, most likely, many of such systems will find application for solving quite a certain class of problems. A clear illustration of this is the use of tunable focusing systems to support the operation of groups of UAVs. Nevertheless, a pronounced competition between different directions of development of signal processing systems seems inevitable. The authors are of the opinion that, over time, a certain mainstream idea [11] will be formed that suppresses alternative points of view. An example of this is a historical fact: binary logic, which is the basis for the functioning of computing machines, displaced ternary logic for many decades, despite many advantages of the latter [12,14]. It is not excluded, however, that there will be several such dominating directions.

There is also no doubt that such competition will have an economic background related to the interests of producers, i.e., it will be largely spontaneous. The alternative is a purposeful choice of the most preferable options. Consequently, the development of prognostic models of computer technology development (signal and image processing systems in general) becomes urgent, and there are prerequisites for this. In particular, it is already possible to raise the question about the predictive choice of the algorithmic basis of future computer technology, and further, to select the most suitable one from the available set.

This choice will be inseparably connected with the choice of element base, and, consequently, with the choice of materials on the basis of which it can be realized. Consequently, it is reasonable to raise the question about the complementary development of the whole direction of polymer sciences, which is connected with the creation of neuromorphic/learning systems and those sections of information technologies, which operate with multivalued logic. Polymer sciences have long been based on the thesis of creating materials with predetermined properties. There is every reason to extend this thesis to the creation of neuromorphic/learning systems, based on the premise that they can realize almost any learning algorithm.

## 5. Conclusions

The prospects of using systems based on hydrophilic polymers for signal processing are more than extensive. These prospects are related both to the creation of a quasi-biological alternative for existing computational systems, largely focused on the von Neumann architecture, and to the creation of neuromorphic systems for various purposes. The latter include, in particular, the creation of controllable systems that are a “hybrid” of holograms and neural networks, allowing us to realize new approaches to processing not only signals but also images. It was obviously impossible to cover the whole range of works carried out in this direction in a separate review, but we did not pursue such a goal.

We aimed to show that the use of hydrogels as a basis for composite materials intended for signal processing (including the need for further improvement of computing technology) will inevitably put problems before researchers of a purely interdisciplinary nature.

Even a superficial analysis of this problem convinces us that it is necessary to increase the level of interdisciplinary cooperation between the developers of analogues of computational systems based on organic materials (in particular, polymer hydrogels) and specialists in the field of physical optics, applied abstract algebra, etc. In particular, the materials of this review show that the well-known thesis “polymer science allows to obtain functional materials with predetermined properties” can and should receive a new sounding. It is reasonable to raise the question of creating organic-based materials that will realize certain algorithms of digital signal and image processing (including computational operations), which will meet the specific needs of practice.

## Data Availability

The original contributions presented in the study are included in the article; further inquiries can be directed to the corresponding author.
